# Analysis of periplasmic sensor domains from *Anaeromyxobacter dehalogenans* 2CP-C: Structure of one sensor domain from a histidine kinase and another from a chemotaxis protein

**DOI:** 10.1002/mbo3.112

**Published:** 2013-07-30

**Authors:** P Raj Pokkuluri, Jeff Dwulit-Smith, Norma E Duke, Rosemarie Wilton, Jamey C Mack, Jessica Bearden, Ella Rakowski, Gyorgy Babnigg, Hendrik Szurmant, Andrzej Joachimiak, Marianne Schiffer

**Affiliations:** 1 Biosciences, Argonne National Laboratory9700 South Cass Avenue, Lemont, Illinois, 60439; 2 Structural Biology Center, Biosciences division, Argonne National LaboratoryLemont, Illinois, 60439; 3 The Midwest Center for Structural Genomics, Biosciences division, Argonne National Laboratory9700 South Cass Avenue, Lemont, Illinois, 60439; 4 Department of Molecular and Experimental Medicine, The Scripps Research Institute10550 N Torrey Pines Rd, La Jolla, California, 92037

**Keywords:** Acetate, chemotaxis, helical bundle, PAS-like, periplasmic sensor domains, sensor histidine kinase

## Abstract

*Anaeromyxobacter dehalogenans* is a δ-proteobacterium found in diverse soils and sediments. It is of interest in bioremediation efforts due to its dechlorination and metal-reducing capabilities. To gain an understanding on *A. dehalogenans'* abilities to adapt to diverse environments we analyzed its signal transduction proteins. The *A. dehalogenans* genome codes for a large number of sensor histidine kinases (HK) and methyl-accepting chemotaxis proteins (MCP); among these 23 HK and 11 MCP proteins have a sensor domain in the periplasm. These proteins most likely contribute to adaptation to the organism's surroundings. We predicted their three-dimensional folds and determined the structures of two of the periplasmic sensor domains by X-ray diffraction. Most of the domains are predicted to have either PAS-like or helical bundle structures, with two predicted to have solute-binding protein fold, and another predicted to have a 6-phosphogluconolactonase like fold. Atomic structures of two sensor domains confirmed the respective fold predictions. The Adeh_2942 sensor (HK) was found to have a helical bundle structure, and the Adeh_3718 sensor (MCP) has a PAS-like structure. Interestingly, the Adeh_3718 sensor has an acetate moiety bound in a binding site typical for PAS-like domains. Future work is needed to determine whether Adeh_3718 is involved in acetate sensing by *A. dehalogenans*.

## Introduction

Bacteria employ a variety of signal transduction proteins to sample their environment and initiate appropriate cellular responses. The most heavily utilized bacterial tools for signal transduction are the so-called two-component signal transduction systems (reviewed in Bourret and Silversmith 2010). The prototypical two-component system consists of a sensor histidine kinase (HK) and a response regulator. Paired proteins communicate via phosphoryl group transfer thereby connecting a stimulus that is detected by the HK protein with a cellular response mediated by the response regulator, which most commonly acts as a transcription factor. The chemotaxis pathway that mediates directed motility in response to environmental conditions represents a modification of this simple scheme. Here, receptor and kinase activities are encoded on two separate polypeptides, the methyl-accepting chemotaxis proteins (MCP's) and the cytoplasmic CheA kinase, respectively. In analogy to the prototypical two-component system, CheA communicates with a response regulator protein CheY by phosphoryl group transfer and CheY regulates motility by interaction with the motility apparatus rather than by acting as a transcription factor (Szurmant and Ordal 2004; Wadhams and Armitage 2004).

**Figure 1:**
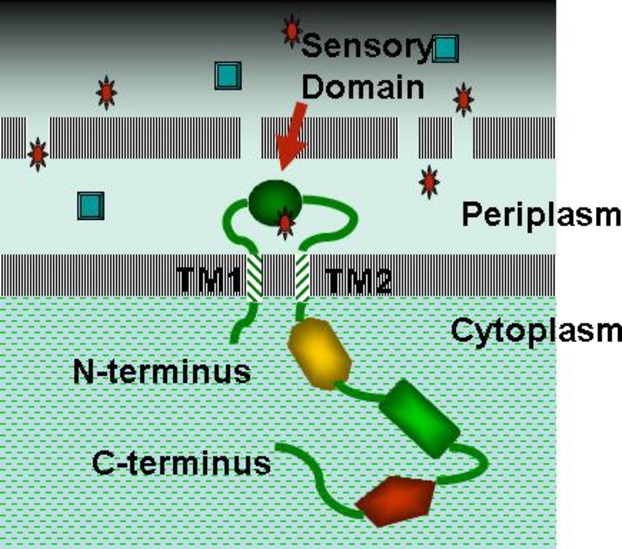
Schematic drawing of a typical transmembrane signal transduction protein in a cell. The cytoplasmic output domains are shown in different colors and shapes to indicate that they vary depending upon the nature of the signal transduction protein (HK or MCP).

Many of the HK and MCP signal transduction proteins span the cytoplasmic membrane and detect extracytoplasmic signals/stimuli via “sensor” domains that face the periplasm (reviewed in Szurmant et al. 2007). Binding of a small molecule or other stimulus to these domains initiates a response in the cytoplasmic domains, which in turn triggers the downstream regulatory processes (Fig. 1). The exact “stimulus” detected by the periplasmic sensor domains in many cases is not known (for reviews see Cheung and Hendrickson 2010; Moglich et al. 2009). The stimulus could be a ligand binding or change in pH or change in reduction potential etc.

*Anaeromyxobacter dehalogenans* is a bacterium widely found in soils and sediments and exhibits metabolic and respiratory versatility. It grows under various redox conditions and can couple the oxidation of variety of substances, such as formate, acetate, hydrogen etc. to the reduction of different electron acceptors such as U(VI), Fe(III), halogenated phenols etc. (Sanford et al. 2002; He and Sanford 2003; Wu et al. 2006). These characteristics make *A. dehalogenans* of interest in bioremediation efforts. The sequence of *A. dehalogenans* strain 2CP-C genome was determined at the DOE Joint Genome Institute (Thomas et al. 2008) but few studies investigating its proteins have emerged.

A likely contributing factor to the ability of *A. dehalogenans* to thrive in contaminated environments is its complement of signal transduction proteins. A measure to evaluate and compare signal transduction capabilities between different bacteria, the “bacterial IQ” was introduced by Michael Galperin (http://www.ncbi.nlm.nih.gov/Complete_Genomes/SignalCensus.html; Galperin et al. 2010). Accordingly, *A. dehalogenans* has an IQ of 134 suggestive of its complex lifestyle and adaptability to harsh environmental challenges.

We have undertaken a study of the periplasmic sensor domains from *A. dehalogenans* 2CP-C with an eventual goal of delineating the functions of some of the signal transduction proteins (The term “signal transduction proteins” hereafter is used to refer to transmembrane sensor histidine kinases [HK] and methyl-accepting chemotaxis [MCP] proteins only) from this organism. According to the Microbial Signal Transduction (MiST) database (Ulrich and Zhulin 2007) *A. dehalogenans* 2CP-C has 76 HK, and 18 proteins annotated as MCP. We found that among these 24 HK and 12 MCP proteins have a periplasmic sensor domain (see Table 1). We predicted the structures of the sensor domains and have undertaken a study to characterize them by cloning, expression, isolation, crystallization, and structure determination. In principle, characterization of the sensor domains of the signal transduction proteins could contribute to identification of the molecular stimuli detected by these transmembrane receptors, which should aid in understanding of the functions of the corresponding signal transduction proteins.

**Table 1:** Predicted structural folds for periplasmic sensor domains from *A. dehalogenans* 2CP-C

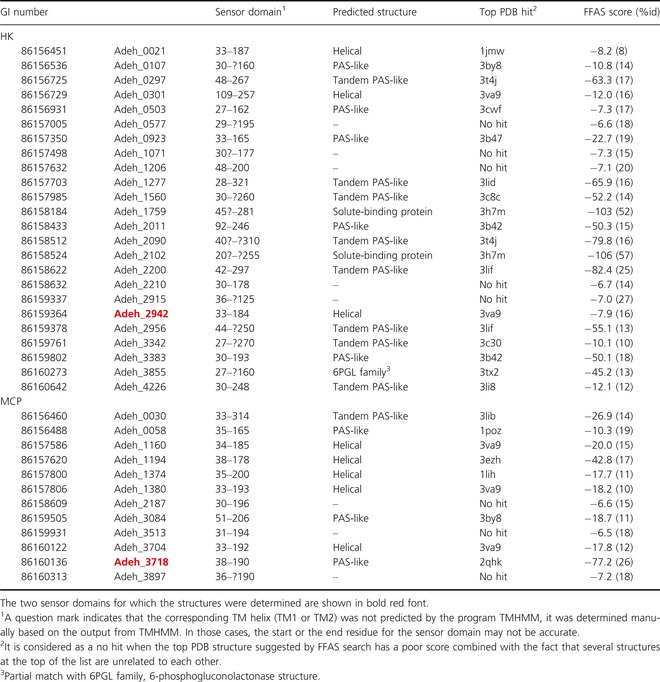

## Experimental Procedures

### Identification of the periplasmic sensor domain segments and prediction of their structure

The signal transduction proteins listed for *A. dehalogenans* 2CP-C in the Microbial Signal Transduction Database, MiST (2007) (Ulrich and Zhulin 2007) was searched with keywords HisKA (sensor histidine kinase domain) and MCP (methyl-accepting chemotaxis domain, also known as MA domain). By visual inspection we selected sequences that had about 100–300 residues between two predicted N-terminal transmembrane helices or long hydrophobic segments that could represent globular periplasmic sensor domains. The MiST database provides both the SMART (Letunic et al. 2012) and Pfam (Finn et al. 2010) annotations for each of the two-component system proteins. In most cases, we used the annotations from SMART instead of Pfam as the SMART annotation identified more transmembrane (TM) helices or hydrophobic segments. The program TMHMM (Krogh et al. 2001) was used to determine the positions of the two TM helices which were then used to define the starting and ending residues for each of the periplasmic sensor domains. The structures of the sensor domains were predicted using the Fold & Function Assignment System (FFAS) server (Jaroszewski et al. 2011). Table 1 shows the potential periplasmic sensor domains identified in each of the signal transduction proteins of *A. dehalogenans* including the prediction of their structures. As noted in Table 1, the boundaries of some of the TM helices were estimated manually because in those cases the TM helix prediction probability was not 100% and therefore the TMHMM program did not define them as TM helices.

### Cloning, expression, and purification

The periplasmic sensor domains of *A. dehalogenans* 2CP-C listed in Table 1 (except Adeh_0030, Adeh_0058, Adeh_1160, and Adeh_3704) were amplified by polymerase chain reaction (PCR) from genomic DNA with KOD Hot Start DNA polymerase (EMD Millipore, Darmstadt, Germany) using appropriately designed forward and reverse primers. The PCR products were purified (QIAquick PCR Purification Kit, Qiagen), treated with T4 DNA polymerase in the presence of Deoxycytidine triphosphate, cloned into the pMCSG7 vector (Donnelly et al. 2006) according to the ligation-independent procedure (Aslanidis and de Jong 1990; Eschenfeldt et al. 2009) and transformed into *E. coli* BL21(DE3)-Gold (Stratagene), which harbors an extra plasmid, pMgk, encoding one rare tRNA corresponding to rare Arg codons, AGG and AGA. To produce the proteins, bacterial cultures were grown at 37°C, 190 rpm in 1 L of enriched M9 medium (Donnelly et al. 2006) until an OD_600_ of 1.0 was reached. After air cooling the culture at 4°C for 60 min, 90 mg/L of selenomethionine (SeMet) and 25 mg each/L of the inhibitory amino acids l-valine, l-isoleucine, l-leucine, l-lysine, l-threonine, and l-phenylalanine (Medicillin, Inc., catalog number MD045004D) were added. Subsequently, protein expression was induced with 0.5 mmol/L isopropyl-β-D-thiogalactoside (IPTG), and the incubator temperature was increased to 18°C. Following overnight incubation, the cells were harvested by centrifugation and resuspended in lysis buffer (500 mmol/L NaCl, 5% (v/v) glycerol, 50 mmol/L HEPES pH 8.0, 20 mmol/L imidazole, and 10 mmol/L β-mercaptoethanol). Cells were disrupted by lysozyme treatment (1 mg/mL) and sonication, and the insoluble cellular material was removed by centrifugation. The SeMet-labeled protein was purified from other contaminating proteins using nickel-nitrilotriacetic acid (Ni-NTA) affinity chromatography on an ÄKTAxpress system (GE Healthcare Life Sciences, Piscataway, NJ) as described previously (Kim et al. 2004). The His_6_-tag was cleaved using recombinant, His_6_-tagged Tobacco etch virus protease. This was followed by Ni-NTA affinity chromatography to remove the protease, uncut protein, and affinity tag. The pure protein was concentrated using Amicon Ultra-15 concentrators (Millipore, Bedford, MA) into 20 mmol/L HEPES pH 8.0 buffer, 250 mmol/L NaCl, and 2 mmol/L dithiothreitol (DTT).

### Crystallization

The purified and concentrated sensor domain proteins were used in identification of crystallization conditions. Sitting drop vapor diffusion method using drops composed of 0.4 μL protein plus 0.4 μL well solution were equilibrated against a reservoir of 140 μL of reagent solution. The crystallization plates were setup with the help of the Mosquito liquid dispenser (TTP LabTech, Cambridge, MA). Preliminary conditions identified for Adeh_2942 and Adeh_3718 sensor domains with MCSG screens 1–4 (Microlytic Inc., Burlington, MA) were further optimized by inclusion of similar reagents from other commercial screens. The crystals used for data collection were grown by hanging drop vapor diffusion.

Adeh_2942 sensor was crystallized from Wizard II reagent #11 (10% 2-propanol, 0.1 mol/L sodium cacodylate pH 6.5, 0.2 mol/L zinc acetate). Crystal was cryoprotected by transfer to a solution containing 75 μL reservoir solution and 25 μL ethylene glycol for 15–30 sec prior to plunging in liquid nitrogen.

Adeh_3718 sensor was crystallized from Membfac (Hampton Research) reagent #2 (12% PEG4000, 0.1 mol/L sodium acetate pH 4.6, 0.1 mol/L zinc acetate) diluted by water as follows: 350 μL reagent solution + 150 μL deionized water. Cryoprotection was achieved by supplementing the reservoir solution with ethylene glycol as described above for the Adeh_2942 sensor.

### Data collection and structure determination

X-ray diffraction data were collected at the selenium edge at the Structural Biology Center 19-BM (Advanced Photon Source, Argonne, IL). Data were processed with the program HKL3000 (Minor et al. 2006). Both structures were determined by the single wavelength anomalous dispersion (SAD) method using the automated procedures for structure solution, phasing and auto building of the model into electron density maps within the HKL3000 package. Refinements of the structures were initially carried out with refmac5 (Murshudov et al. 2011) and in the final stages with Phenix (Adams et al. 2010). The data collection parameters and final refinement statistics are presented in Table 2.

**Table 2:** Crystallographic parameters and refinement statistics

	Adeh_3718	Adeh_2942
Crystal & data parameters		
*a* (Å)	50.595	87.693
*b* (Å)	50.595	75.184
*c* (Å)	112.733	51.031
*α* (°)	90.0	90.0
*β* (°)	90.0	105.18
*γ* (°)	120.0	90.0
Space group	P3_1_21	C2
#mol/AU	1	2
V_M_ (% solvent)	2.8 (56)	2.6 (53)
Wavelength (Å)	0.97911	0.97903
Resolution^1^ (Å)	100–2.0 (2.02–2.00)	100–2.0 (2.03–2.00)
R-merge^1^ (%)	3.0 (19.6)	6.1 (36.0)
Redundancy^1^	5.8 (4.6)	2.3 (1.8)
Completeness^1^ (%)	99.9 (100)	90 (62)
Mean I/σ(I)^1^	53 (8)	18 (2)
Refinement		
Program used	Phenix	Phenix
Resolution range (Å)	35–2.0	30–2.0
No. of reflections	21,799	37,771
R-factor	0.195	0.198
R-free^2^	0.238	0.251
Protein atoms (mean *B*-factor, Å^2^)	1144 (40.0)	2060 (38.5)
Solvent atoms^3^ (mean *B*-factor, Å^2^)	81 (37.8)	229 (45.2)
Rmsd bonds (Å)	0.019	0.018
Rmsd bond angles (°)	1.7	1.7
PDB accession code	4K08	4K0D

1 Values in parentheses are for the highest resolution shell.

2 Calculated from a random set of reflections (5%) not used in refinement.

3 Includes water and other ions refined in the structure.

## Results

### Periplasmic sensor domains

We have examined the sequences of signal transduction (HK and MCP) proteins from *A. dehalogenans* strain 2CP-C in the MiST database (Ulrich and Zhulin 2007) to identify proteins that have periplasmic sensor domains. We looked for proteins containing 100–300 residues positioned between the two N-terminal transmembrane helices (see Fig. 1). According to these definitions, 12 of the 18 chemotaxis proteins and 24 of the 76 sensor histidine kinases have periplasmic sensor domains (Table 1). The boundaries of these periplasmic domains were decided based on the prediction of the TM helices in these proteins.

When utilizing the fold prediction server FFAS, a majority of the *A. dehalogenans* sensor domains were predicted to be either helical, or Per-Arnt-Sim (PAS)-like or tandem PAS-like domains (Table 1). For the HK sensors, three were predicted to be helical, five PAS-like, seven tandem PAS-like; interestingly two HK sensors (Adeh_1759 and Adeh_2102) were predicted to be homologous to bacterial periplasmic solute-binding protein, and another (Adeh_3855) predicted to have a partial match to structure of 6-phosphogluconolactonase. For five HK sensor sequences there was no reliable prediction (poor FFAS score combined with the fact that several structures at the top of the list were unrelated to each other). Among the twelve MCP sensors, five were predicted to be helical, three PAS-like, one tandem PAS-like; for three sequences there was no reliable prediction. Three sensors (Adeh_0923, Adeh_2011, and Adeh_3383) had PAS-like folds homologous to sensor domains of MCP proteins from *Geobacter sulfurreducens*, GSU0935, and Gsu0582 (Protein Data Bank [PDB] codes, 3b47 and 3b42; Pokkuluri et al. 2008) containing a *c*-type heme-binding sequence motif located in similar positions; Adeh_2011 and Adeh_3383 have an additional heme-binding motif as also observed in case of *G. sulfurreducens* proteins (Gsu0591, Gsu0599; Londer et al. 2006).

Thirty two sensor domains were included in a high throughput cloning and purification pipeline of the Midwest Center for Structural Genomics (Argonne National Laboratory, IL). Based on the expression levels and solubility tests (data not shown) under standard expression conditions, seven sensor domains, four from HK proteins (Adeh_1071, 2090, 2210, 2942) and three from MCP proteins (Adeh_1380, 3718, 3897) were selected for large-scale protein preparation. The sensor domains were produced in conditions that incorporate selenomethionine for structure determination by multiple wavelength anomalous dispersion (MAD) method. Crystallization trials were carried out for all eight purified sensor domains. Hit conditions were identified for three sensor domains (Adeh_2942, Adeh_3718, and Adeh_2187). Optimizations of the hit conditions have led to data collection quality crystals for two (sensor domain of HK protein, Adeh_2942 and sensor domain of MCP protein, Adeh_3718) followed by their structure determination by X-ray crystallography. The structures of the two sensor domains are described below.

### Structure of Adeh_2942 sensor domain

Periplasmic sensor domain (residues 33–184) of chemotaxis protein Adeh_2942 was isolated, purified, and crystallized as described in the methods section. Adeh_2942 crystallized in space group C2 and diffracted X-rays to 2 Å resolution. Structure was determined by SAD method utilizing the selenomethionine residues and refined to an R-factor of 0.198 and R-free of 0.251. The crystallographic data and refinement statistics are presented in Table 2. Electron density is observed from residues 37 to 179 in monomer A and residues 37 to 181 in monomer B.

**Figure 2:**
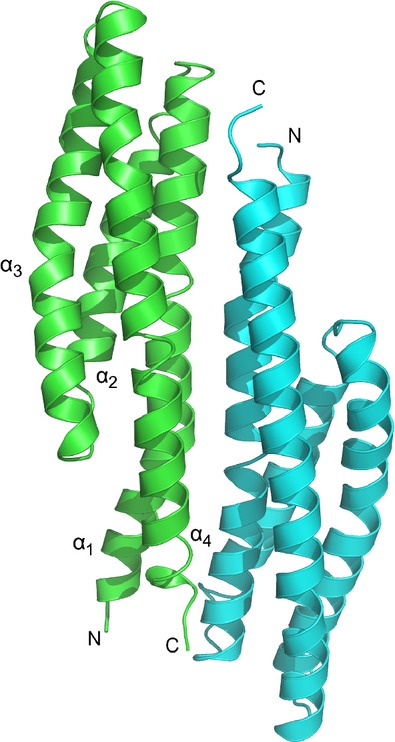
A cartoon representation of the structure of Adeh_2942 sensor domain. A head-to-tail dimer structure is observed in the crystals. The Cα tracing of monomer A is shown in green and that of monomer B is shown in cyan. The helices of monomer A are labeled.

The sensor domain of Adeh_2942 crystallized as a head-to-tail dimer comprised of monomers that form up-down four-helix bundles (see Fig. 2). The root mean square deviation (rmsd) between the two monomers is 0.5 Å when 142 C_α_ atoms are overlapped. The four helices are formed by residues 38–75 (α_1_), 77–101 (α_2_), 104–134 (α_3_), and 137–179 (α_4_) (see Fig. 2). Helices 1 and 4 are longer, whereas helices 2 and 3 are somewhat shorter. The helices α_1_ and α_4_ have a kink at residues Glu46 and Ala146, respectively. The N- and C-termini of the sensor domain are juxtaposed so they can attach to the TM helices as expected in the full-length protein.

### Structural homology of Adeh_2942

DALI structural homology search (Holm and Rosenström 2010) performed with the monomer labeled A of Adeh_2942 has returned several helical bundle sensor domains from PDB. The top hit is a sensor domain of histidine kinase HK9 from *Rhodopseudomonas palustris* of an unknown function (PDB code, 3va9; Z score = 14.7; rmsd 1.9 Å over 122 C_α_ atoms) and the second one is the sensor domain of aspartate receptor, Tar (Bowie et al. 1995; PDB code, 2asr; Z score = 14.5; rmsd 1.9 Å over 130 C_α_ atoms) followed by the serine receptor Tsr sensor domain (Tajima et al. 2011; PDB code, 3atp; Z score = 13.9; rmsd 1.9 Å over 135 C_α_ atoms).

### Structure of Adeh_3718 sensor domain

Periplasmic sensor domain (residues 38–190) of chemotaxis protein, Adeh_3718 was isolated, purified, and crystallized as described in the methods section. Adeh_3718 crystallized in space group P3_1_21 and diffracted X-rays to 2 Å resolution. The structure was determined by the SAD method utilizing the selenomethionine residues and refined to an R-factor of 0.195 and R-free of 0.238. The crystallographic data and refinement statistics are presented in Table 2. Electron density is observed from residues 38 to 181. The crystals consist of one protein molecule per asymmetric unit and there is no indication of oligomeric interactions in the crystal as determined by the PISA server (Krissinel and Henrick 2007).

**Figure 3:**
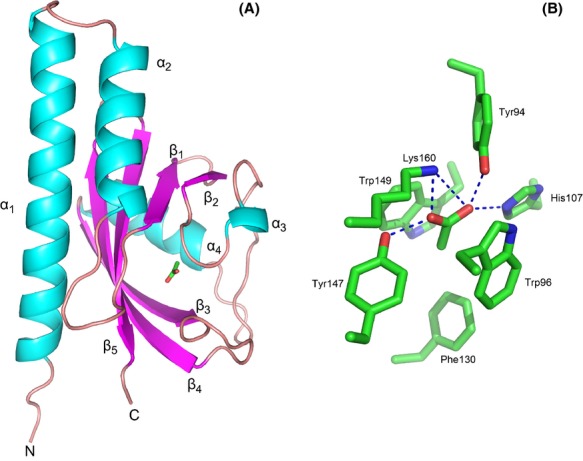
(A) A cartoon representation of the overall structure of Adeh_3718 sensor domain. Note the acetate ion bound in a typical binding site similar to binding of small molecules observed in various other PAS-like domains. (B) A close up view of the acetate-binding site as observed in the crystal structure of Adeh_3718 sensor domain.

The PAS-like domain of Adeh_3718 (Fig. 3A) consists of two N-terminal helices α_1_ (residues 41–68), α_2_ (residues 72–87), followed by two small β-strands β_1_ (residues 96–100) and β_2_ (103–106), helix α_3_ (residues 110–114), helix α_4_ (residues 128–141), β_3_ (residues 142–151), β_4_ (residues 156–167), and β_5_ (residues 171–180). The structure of Adeh_3718 sensor has revealed a fully occupied acetate ion moiety bound to the protein in this binding site (Fig. 3B).

### Acetate-binding site

The binding site is well optimized for an acetate ion as illustrated by the interactions shown in Figure 3B. The acetate ion is bound stacked between two tryptophan rings of Trp96 and Trp149. Salt bridges are observed between the carboxylate group of acetate ion and side chains of Lys160 and His107. Two additional hydrogen bonds are made with the side chains of Tyr94 and Tyr147. The methyl group of the acetate is facing a hydrophobic pocket lined with the side chains of Leu129, Phe130, and Phe133.

### Structural homology of Adeh_3718

Structural homology search with the program DALI has returned a number of PAS-like sensor domain structures from PDB. The top two closest hits are a sensor domain of an MCP protein of unknown function from *Vibrio parahaemolyticus* RIMD 2210633 (PDB code, 4exo; Z score = 20.0; rmsd 1.9 Å over 137 C_α_ atoms) and a sensor domain of chemoreceptor TlpB from *Helicobacter pylori* involved in pH sensing (Sweeney et al. 2012; PDB code, 3ub8; Z score = 17.6; rmsd 2.2 Å over 136 C_α_ atoms).

## Discussion

### Periplasmic sensor domains of *A. dehalogenans* 2CP-C

We analyzed all the possible periplasmic sensor domains of signal transduction proteins from *A. dehalogenans* strain 2CP-C. The sensor domains are part of signal transduction systems of HK and MCP proteins; they are located in the periplasm flanked by two transmembrane segments as shown in the schematic (Fig. 1). We determined the boundaries of the sensor domains by determining the termini of the transmembrane helices. We used the FFAS server to predict their structures. Based on this analysis the domains belong to one of several classes (as shown in Table 1): (1) All α-helical structures similar to the aspartate chemotaxis receptor, Tar (Bowie et al. 1995), (2) PAS-like domains similar to proteins such as the citrate receptor, CitA (Reinelt et al. 2003) and heme containing PAS-like domains that we previously studied from *G. sulfurreducens* (Pokkuluri et al. 2008), (3) Tandem PAS-like domains such as in KinD (Wu et al. 2013), (4) Two sensor domains with a solute-binding protein fold as previously identified by Cheung et al. (2009), and (5) One sensor domain predicted to be homologous to 6-phosphogluconolactonase (Delarue et al. 2007), a fold not previously observed for a sensor domain. For eight of the sensors there were no predictions of homologous structures that appeared to be significant. It is possible that these sequences could represent one or more new folds yet to be observed for sensor domains of HK or MCP proteins.

**Figure 4:**
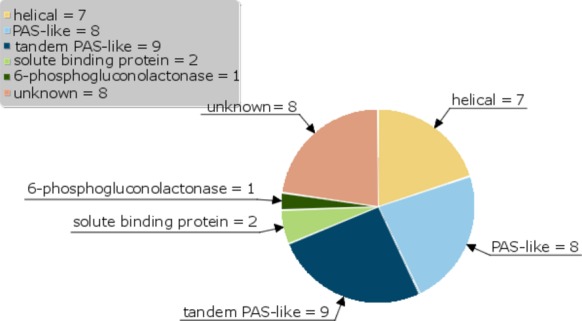
A Pie chart representation of the tertiary folds predicted (results from Table 1) for the periplasmic sensor domains of *A. dehalogenans* 2CP-C.

The tertiary fold predictions of the sensor domains from *A. dehalogenans* are shown in a pie chart representation (Fig. 4). It is interesting to note that for chemotaxis proteins there is roughly an equal number of helical and PAS-like sensors, whereas for HK proteins there is a clear bias toward PAS-like sensor domains. It is not clear whether this is broadly applicable or it may be coincidental and limited to this organism. However, a previous analysis of HK proteins from *B. subtilis*, *E. coli,* and *S. aureus* also implicated the PAS-like domain as the dominant periplasmic sensing fold for HK in these organisms (Chang et al. 2010).

Two of the sensor domains (Adeh_3718 and Adeh_2942) were crystallized and their structures determined by X-ray diffraction. In both cases, the structures confirmed the predictions from the corresponding sequences.

### Adeh_2942 sensor

**Figure 5:**
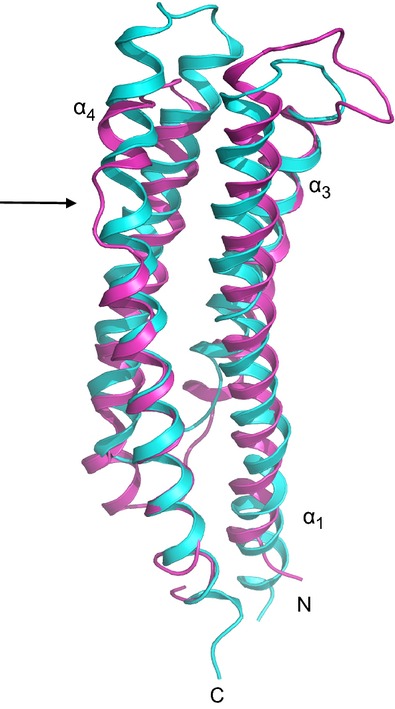
Cartoon representation of helical bundles formed by the monomer of Adeh_2942 sensor and that of Tar are shown. The aspartic acid binding site in Tar is indicated by an arrow. Please note the significant deviation from helical geometry at the binding site in the helix α_4_ in Tar. Adeh_2942 is shown in cyan and Tar in pink. The two structures were overlapped by the SSM routine in Coot (Emsley et al. 2010).

No significant matches were found in the Pfam database for the sensor domain of Adeh_2942, however, FFAS03 server predicted it to be helical. Indeed, the crystal structure of Adeh_2942 revealed a helical bundle. In general, the sensor domains of signal transduction proteins have either weak or no oligomeric association in solution. In case of Adeh_2942 sensor, the crystallization process selected a head-to-tail dimer; in contrast the well-studied sensor domains of chemotaxis proteins, aspartate receptor, Tar (Bowie et al. 1995), serine receptor, Tsr (Tajima et al. 2011), and the nitrate sensing histidine kinase, NarX (Cheung and Hendrickson 2009) form head-to-head dimers that are considered physiologically relevant, and the ligand in each case is bound between the two monomers. The four-helix bundle structure of the monomer observed in Adeh_2942 is similar to that of Tar and Tsr sensor domains. An overlay of the helical bundles formed by the monomers of Adeh_2942 sensor and Tar are shown in Figure 5. The ligand-binding site in Tar and Tsr is located near residues that deviate from the helical secondary structure (form a kink) in helix-4 that includes a proline (residues Gln152 and Pro153 in Tar, and residues Phe152, Asp153, Gln154, and Pro155 in Tsr; binding site in Tar is indicated by an arrow in Fig. 5). Comparison of the monomer structure of Adeh_2942 sensor domain with that of Tar and Tsr has revealed a minor deviation from helical secondary structure in helix-4 (residues Ala146 and Thr147) in approximately similar location, however, the two prolines present in Adeh_2942 (residues 149 and 150) are within the helical secondary structure. The structure-based sequence alignment (not shown) between Adeh_2942, Tar, and Tsr have not revealed any significant clues as to the nature of the binding site in Adeh_2942 sensor.

### Adeh_3718, a possible acetate sensor

The Adeh_3718 sensor domain from an MCP protein forms a PAS-like fold as predicted. The PAS-like fold was originally reported for periplasmic sensor domain of citrate receptor, CitA (Reinelt et al. 2003) and for fumarate sensor, DcuS (Pappalardo et al. 2003). This domain was referred to as PAS-like by Hefti et al. (2004) where the authors have noted that the tertiary fold of these extracellular sensor domains was similar but not identical to the classic PAS fold observed in cytoplasmic sensor domains. Later this fold was observed in periplasmic sensor domain of a HK protein, PhoQ (Cho et al. 2006; Cheung et al. 2008) and in periplasmic sensor domains of chemotaxis proteins, GSU0935 and GSI0582 (Pokkuluri et al. 2008). Cheung et al. (2008) named this domain fold as PDC (PhoQ-DcuS-CitA) sensor fold. The domain fold is characterized by an N-terminal helix-loop-helix followed by an antiparallel generally five strand β-sheet, which may be surrounded by additional helices on either side (Fig. 2). In general, the N-terminal helix-turn-helix does not contribute to the binding site. The small molecule binding residues come from β-strands, loops connecting β-strands, and from the helical fragments other than the N-terminal helices.

Residues 37–131 of Adeh_3718 sensor domain were predicted to form a Cache-2 domain by SMART (Letunic et al. 2012) and Pfam (Punta et al. 2012) databases. Cache is a signaling domain observed in various Ca^2+^ channel subunits and chemotaxis receptors and was suggested to be involved in small molecule binding (Anantharaman and Arvind 2000). These authors point to a conserved histidine in the C-terminal part of Cache (Cache-C) and suggested its involvement in binding the small molecule by this domain. Indeed, His107 in Adeh_3718 sensor which corresponds to the residue pointed out by these authors, interacts with the acetate ion (see Fig. 3B). It is also interesting that the predicted Cache-2 domain of Adeh_3718 sensor actually turns out to have a PAS-like fold, whereas the periplasmic domains of CitA_sensor and DcuS_sensor that also form a PAS-like fold are predicted as Cache-3 domains.

**Figure 6:**
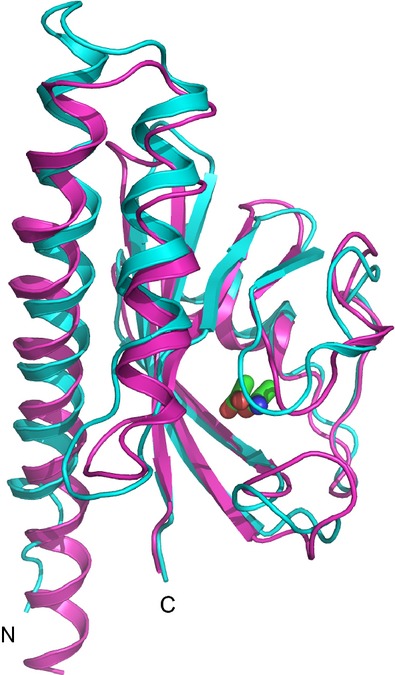
An overlap of the Adeh_3718 sensor domain with bound acetate and the sensor domain of TlpB complexed with acetamide (PDB code, 3ub7) is shown. The two structures were overlapped with SSM routine in Coot. Adeh_3718 sensor is shown in cyan and TlpB sensor in pink. The bound small molecules acetate and acetamide are shown in ball-stick representation with atom type colors (carbon: green, oxygen: red, nitrogen: blue). Note the similarity of the two structures and the binding sites. The TlpB sensor has an additional helix at the C-terminus (residues 188–201) that is not present in Adeh_3718 sensor; this helix is not shown in the figure for clarity purposes.

Adeh_3718 sensor domain structure has revealed a fully occupied acetate ion. The acetate ion is bound in a typical binding site observed for the PAS-like domains, such as CitA and DcuS, where the physiological ligands are known to bind. A molecule of urea, suggested as being involved in pH-sensing by the TlpB sensor, is bound in a similar binding site (Sweeney et al. 2012). For comparison purposes, an overlay of the structures of the Adeh_3718 sensor with bound acetate and TlpB sensor complexed with acetamide (PDB code, 3UB7) are shown in Figure 6. In case of the structure of MCP sensor from *Vibrio parahaemolyticus* (the top hit from DALI search with Adeh_3718 sensor), 4exo, the authors have modeled a pyruvate molecule in a similar binding site. In addition, in case of tandem PAS-like domains, the membrane-distal PAS-like domain also shown to bind small molecules in similar binding sites (Zhang and Hendrickson 2010; Wu et al. 2013). Interestingly, an R95A mutant of KinD sensor binds acetate ion in the membrane-distal PAS-like domain, whereas the wild type protein binds pyruvate (Wu et al. 2013). It appears that small carboxylic acids are common ligands accommodated by the periplasmic PAS-like sensors.

The binding site observed in Adeh_3718 can accommodate a formate or a propionate moiety but accommodation of larger monocarboxylic acids is doubtful. It is unknown whether the sensor domain picked up the acetate ion during expression in *E. coli* or during crystallization in conditions containing acetate (sees methods section). Although acetate is a frequently used electron donor for organisms such as *A. dehalogenans*, a chemoreceptor for acetate has not been reported. He and Sanford (2004) have demonstrated the ability of *A. dehalogenans* to compete for nanomolar level acetate as the electron donor under different growth conditions. Although PAS-like domains can bind ligands nonspecifically (Airola et al. 2010) and acetate binding by Adeh_3718 sensor may just be a crystallization artifact, one cannot rule out the possibility that Adeh_3718, which is a chemotaxis protein, could be involved in sensing environmental acetate by *A. dehalogenans*. Only future work can determine for sure whether or not Adeh_3718 is involved in acetate sensing by *A. dehalogenans*.

Physiological stimuli for the histidine sensor kinase, BarA, from *E. coli* was reportedly provided by either formate, acetate, propionate, or other short chain monocarboxylic acids (Chavez et al. 2010). BarA (GI 170082357) has a sensor domain in the periplasm, residues 34–175 in between two TM helices, which we modeled by FFAS03 server (score −22.2) to be similar to periplasmic PAS-like sensor domain of CitA. However, the sequence identity between the sensor domain of Adeh_3718 and that of BarA is very low and none of the residues involved in acetate binding by Adeh_3718 sensor appear to be conserved in the BarA sensor.

The proteome profiling of *A. dehalogenans* 2CP-C during growth with fumarate and ferric citrate was reported (Chao et al. 2010). Although several signal transduction proteins were identified in their experiments, only one protein having a periplasmic sensor domain (subject of our study) was observed. HK protein, Adeh_1759 with a sensor domain having a predicted fold of solute-binding protein (see Table 1) was identified.

Outstanding questions in the field of bacterial signal transduction relate to the identity of the signal and how detection of the signal mechanistically mediates a downstream response, be it by protein regulatory events or chemotaxis. Although sensor domains have been studied for many years, there are still many unknowns about the actual ligands that stimulate a given response and the mechanism of signal transduction. It is now apparent that both HK and MCP proteins interchangeably use the PAS-like (including tandem PAS-like) and helical bundle sensor domain tertiary folds. The main question then is whether the signal transduction mechanism used is specific to a given fold of the periplasmic signal detection domain. If so, is there one activation mechanism for each of the different sensor domain folds? Another pertinent question is how many of the signal transduction proteins directly sense a stimulus and how many require interaction with another protein (for example, LuxQ/LuxP system or recently described chemotaxis sensor of amino acids, McpC from *Bacillus subtilis* (Glekas et al., 2012). Clearly, a lot more experimental work is needed to delineate the functions of sensor domains and fully understand these signal transduction systems.

In summary, we have predicted the structural folds of periplasmic sensor domains of 24 HK and 12 MCP proteins of *A. dehalogenans* strain 2CP-C. All but one of the sensor domains have predicted folds that were previously observed for periplasmic sensor domains, such as helical bundles or PAS-like folds or solute-binding protein fold. Structures of two sensor domains were determined and confirmed their predicted tertiary folds. The Adeh_2942 sensor (HK) was found to have a helical bundle structure, and the other Adeh_3718 sensor (MCP) has a PAS-like structure. Interestingly, the Adeh_3718 sensor has an acetate moiety bound in the typical binding site of PAS-like domains. The possible implications of acetate sensing by this protein await experimental verification.
